# The Binary Bi-Rh Phase Diagram: Stable and Metastable Phases

**DOI:** 10.1007/s11669-017-0600-5

**Published:** 2017-10-13

**Authors:** Peter Kainzbauer, Klaus W. Richter, Herbert Ipser

**Affiliations:** University of Vienna, Faculty of Chemistry, Department of Inorganic Chemistry – Functional Materials, Althanstrasse 14, 1090 Vienna, Austria

**Keywords:** Bi-Rh system, BiRh_0.81_ phase, differential thermal analysis, phase diagram, scanning electron microscopy, x-ray diffraction

## Abstract

The binary bismuth-rhodium (Bi-Rh) phase diagram was reinvestigated from 23 to 60 at.% Rh with focus on the BiRh phase, applying powder-x-ray diffraction (XRD), high temperature powder-XRD, differential thermal analyses and scanning electron microscopy. The phase boundaries of the BiRh phase at 750 °C and the temperature of its peritectic decomposition were refined. In addition, the existence of the two phases Bi_4_Rh and Bi_2_Rh (in two modifications depending on temperature) could be confirmed. Most of the reaction temperatures reported in the literature could be verified within a range of about ± 10 °C. Nevertheless, a few temperatures had to be revised, such as those of the peritectic reactions L + Rh $\rightleftharpoons$ BiRh at 979 °C and L + BiRh $\rightleftharpoons$ *β*-Bi_2_Rh at 785 °C. No evidence could be found for the presence of a stable Bi_3_Rh phase in well annealed samples; from the present results it must be concluded that Bi_3_Rh is actually metastable. On the other hand, a new orthorhombic phase BiRh_0.81_ was discovered which crystallizes in the MnP structure type (Pmna). It was found that the temperatures of the transition between the low-temperature modification *α*-Bi_2_Rh and its high-temperature form *β*-Bi_2_Rh depend considerably on the presence or absence of metastable Bi_3_Rh and stable BiRh_0.81_, respectively.

## Introduction

The intermetallic phase *α*-BiMn with the NiAs-type structure has been suggested as an interesting ferromagnetic material in the past with the major advantage of not containing rare earth elements.[1] Unfortunately, it has not been possible to synthesize this phase as single-phase bulk material despite several decades of intensive research (see e.g. Ref 2-6). A possible approach to circumvent the synthesis difficulties was considered the addition of a third component such as Ni, Pt or Rh, which form intermetallic phases with Bi that are iso-typic to *α*-BiMn.[7-9]

Stabilization of BiMn by Rh was described by Lee et al.[10], however, it was the non ferromagnetic high-temperature modification β-BiMn that was stabilized down to lower temperatures. A ferromagnetic ternary compound Bi_4_Mn_5_Rh_2_ was identified by Street et al.[11] though with a Curie temperature of  −7 °C. A similar observation was made more recently by Taufour et al.[12] who described a ferromagnetic compound BiMn_1.05_Rh_0.02_ with a Curie temperature of 143 °C.

On the other hand, Suits[13] discovered ferromagnetism in Bi-substituted MnRh with the composition Mn_0.8_Bi_0.2_Rh. Based on these observations, a systematic study of the ternary Bi-Mn-Rh system was considered of interest, with the possibility of finding additional intermetallic phases, which might possibly exhibit ferromagnetism. During this study it was found that the binary Bi-Rh phase diagram was still not fully known, in particular, nothing was known about the accurate homogeneity range of the NiAs-type phase BiRh. This triggered a new and thorough investigation of the binary Bi-Rh phase diagram.

In the course of this study it became apparent that some of the phases reported in literature are metastable (as already suspected by other authors), with their appearance depending on the conditions of synthesis and heat treatment. On the other hand, an additional, previously unknown, phase was discovered after extended annealing.

## Literature Review

The first investigations of Bi-Rh alloys date back to Rössler[14] (Bi_4_Rh) and Wöhler and Metz[15] (Bi_2_Rh), and a first phase diagram was presented by Rode.[16] In a series of papers, several Russian research groups (see Ref 17-22 e.g.) studied Bi-Rh alloys with a special focus on superconducting properties. A more refined version of the phase diagram was reported by Kuz´min et al.[21,22] as well as Ross and Hume-Rothery.[9,23] Based on the existing data, Predel[24] assessed the Bi-Rh system. The most recent study of the phase relationships in the Bi-rich part of the Bi-Rh system is from Weitzer et al.,[25] which was included in the most recent assessment of the system by Okamoto.[26]

There are several discrepancies between the published phase diagrams in Ref 24 and 26 especially with regard to the peritectic melting temperatures of *β*-Bi_2_Rh and BiRh, the homogeneity range of BiRh, the existence of a Bi_3_Rh phase, and the transition temperature from *α*-Bi_2_Rh into its high-temperature form *β*-Bi_2_Rh (see Table 1).

**Table 1:** Invariant reactions in the Bi-Rh system

Reaction	Composition, at.% Rh			*T*, °C	Reaction type	Ref.
L + Rh $\rightleftharpoons$ BiRh	46	100	50	977	Peritectic	24
	45	100	< 48	997		26
	***47***	**100**	***50***	**979** **±** **2**		**p.w.**
L + BiRh $\rightleftharpoons$ *β*-Bi_2_Rh	29.5	48	33.3	780	Peritectic	24
	*29.5*	45	33.3	776		26
	***29.0***	***47***	**33.3**	**785** **±** **2**		**p.w.**
L + *β*-Bi_2_Rh $\rightleftharpoons$ Bi_4_Rh	7.0	33.3	20	460	Peritectic	24
	*8.3*	33.3	20	466		26
				**459** **±** **2**		**p.w.**
*β*-Bi_2_Rh $\rightleftharpoons$ *α*-Bi_2_Rh	…	33.3	…	425	Transition	24, 29
	…	33.3	…	370		26
***β*** **-Bi**_**2**_ **Rh** **+** **BiRh**_**0.81**_ $\rightleftharpoons$ ***α*** **-Bi**_**2**_ **Rh**	**<** **33.3**	**45**	**>** **33.3**	**~** **438**	**Peritectoid**	**p.w.**
***β*** **-Bi**_**2**_ **Rh** **+** **BiRh** $\rightleftharpoons$ ***α*** **-Bi**_**2**_ **Rh**	**<** **33.3**	**45**	**>** **33.3**	**448** **±** **2**		
***β*** **-Bi**_**2**_ **Rh** $\rightleftharpoons$ **Bi**_**4**_ **Rh** **+** ***α*** **-Bi**_**2**_ **Rh**	**<** **33.3**	**20**	**>** **33.3**	**426** **±** **3**	**Eutectoid**	
L $\rightleftharpoons$ Bi + Bi_4_Rh	1.0	0.0	20	269	Eutectic	24
	*0.5*	0.0	20	266		26
*α*-Bi_2_Rh + Bi_4_Rh $\rightleftharpoons$ Bi_3_Rh(a)	33.3	20	25	433	Peritectoid(b)	26
Bi_3_Rh $\rightleftharpoons$ *α*-Bi_2_Rh + Bi_4_Rh(a)	25	33.3	20	336	Eutectoid(b)	26

Italics: estimated concentration values; p.w. (bold letters and numbers): present work, if the metastable Bi_3_Rh phase is not present; (a) metastable reactions; (b) reaction not confirmed in this study

The intermetallic phase BiRh has been known to crystallize in the hexagonal NiAs (B8_1_) type structure with space group P6_3_/mmc.[9] This structure type is characterized by atoms of a main group element B (Bi), forming a hexagonal close-packed lattice on Wyckoff position 2*c* (1/3, 2/3, 1/4) with the octahedral sites being occupied by transition metal atoms T (Rh) on Wyckoff position 2*a* (0, 0, 0). In general, the stoichiometry of the NiAs-type phases may vary from TB_2_ to T_2_B, including the ideal composition of TB. A striking feature of NiAs phases is their tendency to form broad homogeneity ranges at high temperatures. The orthorhombic MnP (B31) type structure is closely related to the hexagonal NiAs-type. The MnP structure (see section 4.5 BiRh_0.81_) is a slightly distorted form of the NiAs structure with both B and T atoms on Wyckoff position 4*c* (*x*, 1/4, *z*). (An extensive overview on the properties of NiAs and MnP phases was given by Kjekshus and Pearson[27]).

The compound Bi_2_Rh exists in two modifications, the monoclinic *α* modification, which is stable at room temperature, and the triclinic high-temperature *β* modification. In early studies[18,19,28] the authors described *α*-Bi_2_Rh as a rhombic phase and interpreted a thermal effect at 498 °C as a polymorphic transformation of Bi_2_Rh. This transition temperature was originally estimated by Rode[16] but could not be confirmed by Ross and Hume-Rothery. The latter authors found the transition temperature by high-temperature x-ray diffraction (XRD) at about 430 °C[23] and corrected the temperature in a later publication to 425 °C.[9] These values are in good agreement with thermal arrest measurements by Kuz´min et al. who obtained 420[21] and 430 °C.[22] Kjekshus and Rakke[29] determined the first order transition temperature by DTA analysis and quenching experiments as 425 ± 3 °C, Fjellvag and Furuseth[30] as 427 ± 10 °C. Weitzer et al.[25] found the XRD patterns of the *α*- and *β*-modification of Bi_2_Rh in samples quenched below and above 370 °C, respectively.

Early investigations could not provide single crystals of *β*-Bi_2_Rh of sufficient quality in order to solve the crystal structure. Ruck[31] synthesized *β*-Bi_2_Rh crystals by using the subhalide method and found that the triclinic *β*-phase crystallizes in its own structure type. He pointed out that twinning of *β*-Bi_2_Rh simulates a pseudo-monoclinic cell that is, within error limits, compatible with the structure data of Kuz´min and Zhuravlev.[21] In addition, he concluded that the existence of a monoclinic high temperature γ-modification, emerging from a second order phase transformation, could not be excluded.

The existence of the phase Bi_3_Rh, which was described in Ref 20, 28, 30, 32, and 33 but not detected by Ross and Hume-Rothery,[9] was confirmed by Weitzer et al.[25] These authors described the phase between 433 °C (formed by a peritectoid reaction *β*-Bi_2_Rh + Bi_4_Rh $\rightleftharpoons$ Bi_3_Rh) and 336 °C (eutectoid decomposition into *α*-Bi_2_Rh and Bi_4_Rh). Quite contrary, Fjellvag and Furuseth[30] had reported a transformation of Bi_3_Rh, apparently stable at room temperature, into a two-phase mixture of *α*-Bi_2_Rh and Bi_4_Rh on heating above 172 ± 10 °C.

Apart from these phases, a metastable compound Bi_14_Rh_3_ was reported by Gu et al.[34] It is in metastable equilibrium with the liquid phase and decomposes into Bi_4_Rh and Bi at 247 °C according to Weitzer et al.[25]

Contrary to the Bi-rich part of the Bi-Rh phase diagram, the composition range with *x*_Rh_ > 0.4 is much less extensively investigated. Okamoto[26] gave the stability field of BiRh between 45 and < 48 at.% Rh, independent of temperature. These values were based on data by Glagoleva and Zhdanov[17] who determined the lattice parameters of BiRh as a function of concentration. They annealed the samples at 800 °C, however, they did not quench them but rather cooled them slowly down to room temperature. They found BiRh existing between 44.7 and 47.5 at.% Rh, which indicates a homogeneity range on the Bi rich side of the stoichiometric composition. On the other hand, Ross and Hume-Rothery[9] reported the phase field between 48 and 50.6 at.% Rh based on samples that had been annealed at 780 °C. The same authors measured also the temperature dependence of the lattice parameters for a sample Rh_50_Bi_50_ between room temperature and about 975 °C.

A comparison of crystallographic data available from the literature and data obtained in this study is presented in Table 2.

**Table 2:** Crystallographic data for the Bi-Rh system

Phase	Space group, typ	Lattice parameters							Ref.
		Å			deg			*V*, Å^3^	
		*a*	*b*	*c*	*α*	*β*	*γ*		
Bi_4_Rh	*Ia* $\bar{3}$ *d*	14.928(5)						3326.6	17, 18
		14.9274(2)						3326.2	23
		14.9304(2)						3328.2	25(c)
		14.9290(2)						3327.3	
		14.928						3326.6	28
		**14.930**(**5)**						**3327.9**	**p.w. 430** °C
*α*-Bi_2_Rh	*P2*_*1*_ */c*	6.7	6.8	6.9		117(2)		280.1	20
		6.96(2)	6.83(2)	7.02(2)		118.2		298.4	21
		6.9214(3)	6.7939(4)	6.9601(3)		117.774(3)		289.6	25
		6.805	6.95	6.97		117.80		291.59	29 500 °C
		6.9207(5)	6.7945(4)	6.9613(4)		117.735(6)		289.7	40
		**6.920**(**2)**	**6.794**(**1)**	**6.962**(**2)**		**117.73**(**2)**		**289.76**	**p.w. 400** °C
	orth.	5.90(34)	6.8(3)	7.2(3)				288.8	18, 19, 28
*β*-Bi_2_Rh	*P* $\bar{1}$ *tric*	6.7283(2)	7.0221(3)	7.0655(1)	104.916(2)	100.821(3)	105.832(2)	298.6	25 440 °C
		6.743(1)	7.030(1)	7.067(1)	104.76(1)	100.73(1)	105.79(1)	300.2	31(b)
		**6.738**(**3)**	**7.024**(**7)**	**7.060**(**8)**	**104.81(7)**	**100.70(4)**	**105.77(4)**	**298.7**	**p.w. 730** °C
	mono	15.93(4)	7.04(5)	10.52(3)		92.7		1178.4	21
		16.2(1)	7.0(1)	10.5(1)		92.30		1189.7	18, 19, 28
BiRh	*P6*_*3*_ */mmc*	4.083		5.667				81.817	43
		**4.094**		**5.663**				**82.2**	17 ** 50 at.%Rh**
		4.0894		5.6642				82.03	9
		4.09		5.66				81.99	28
		4.075		5.656				81.33	41
		4.094-4.075		5.663-5.669				82.20-81.52	42 BiRh_0.91_–BiRh_0.81_
		**4.092(2)**		**5.667(5)**				**82.17**	**p.w. 438** °C **Bi**_**50**_ **Rh**_**50**_
**BiRh**_**0.81**_	***Pmna***	**6.175(2)**	**3.782(4)**	**6.550(7)**				**152.95**	**p.w. 440** °C **Bi**_**55**_ **Rh**_**45**_
*Metastable*									
Bi_14_Rh_3_	*Fddd*	6.8959(15)	17.379(3)	31.758(6)				3806.0	34(b)
		6.904(14)	17.3816(9)	31.752(2)				3810.4	25
		7.07(3)	17.55(5)	31.54(8)				3902.2	38(b)
		6.891(1)	17.388(1)	31.1718(2)				3735.0	38
		6.9002(3)	17.3616(7)	31.694(1)				3796.8	39
Bi_3_Rh	*Pnma*	9.0(0.2)	4.2(0.1)	11.4(0.2)				430.92	20(a)
		8.8761(7)	4.2420(3)	11.3627(8)				427.8	25(d)
		8.8718(8)	4.2390(4)	11.365(1)				427.4	25(e)
		8.868(2)	4.240(4)	11.378(4)				427.82	30
		9.027(6)	4.24(2)	11.522(8)				441.0	32
		9.0	4.25	11.4				436.05	28(a)
		9.1	4.2	11.4				435.7	33(a)

**p.w.** = present work, measured at room temperature; (a) denoted as *β*-Bi_4_Rh, Bi_3_Rh was not known at that time; (b) single crystal XRD; (c) different synthesis methods; (d,e) in equilibrium with: traces of Bi(Rh) or Bi_14_Rh_3_ respectively

## Experimental Section

### Sample Preparation

Base material for all samples were pure element pieces of Bi (99.999%, ASARCO, New Jersey, USA, manually pulverized, grain size < 0.09 mm) and Rh powder (99.95% ÖGUSSA, Austria). Calculated amounts of the powders were mixed and pressed to pellets in a 5 mm pressing cylinder under a load of 20-25 kN. Pellets of samples containing more than 52.5 at.% Rh were melted in an arc furnace (Bühler MAM) on a water-cooled copper plate under Ar of 99.999% purity (~ 0.5 bar) using Zr as getter material and sealed into evacuated silica glass tubes (~ 10^−3^ mbar). All other pellets were sealed into evacuated silica glass tubes and melted over an oxyhydrogen flame under shaking, with optical control of the melting process. All samples (total amount 250-1000 mg), were annealed at different temperatures for at least 2 weeks and quenched in cold water. All annealing processes were carried out in muffle furnaces (Nabertherm, Germany) with a temperature accuracy of approximately ± 5 °C.

All alloy compositions given in the Tables are nominal compositions. Independent of the synthesis method, a loss of < 2 wt.% could be determined by re-weighing the quenched samples.

### Characterization

Phase identification was performed at ambient conditions by powder-XRD on a Bruker D8 Advance Diffractometer in Bragg–Brentano pseudo-focusing geometry (reflection setting), using Cu-K*α* radiation and a LynxEye^®^ one-dimensional silicon strip detector (exposure time: 2 h). Two selected samples with 35 and 50 at.% Rh were analyzed by high temperature powder-XRD. The measurement was performed under vacuum, using an Anton Paar XRK 900 reactor chamber on a Bruker D8 Advance Diffractometer. For evaluation and Rietveld refinement of all diffraction patterns the TOPAS^®^ 4.2 software[35] was used.

Samples (except one with 43.5 at.% Rh) containing between 42.5 and 52.5 at.% Rh and annealed at 750 °C were investigated by electron microprobe analysis (EMPA) with a Cameca SX 100 using wavelength dispersive x-ray spectroscopy (WDX) at 20 kV. The instrument was calibrated with Bi and Rh standard material. All other samples were investigated using a scanning electron microscope (Zeiss Supra 55 VP) with energy-dispersive x-ray spectroscopy (EDX). Backscattered electrons were used for surface visualization at 20 kV acceleration voltage. Pure elemental Co and a Bi_50_Rh_50_ standard were applied for energy and intensity calibration. To minimize statistical errors, the average phase composition was obtained from at least eight spot/area scans. A comparison between WDX and EDX measurements on the same samples showed a difference of < 0.8 at.% between the two techniques. On the other hand, the characteristic XRD radiation spectra of Bi and Rh, especially the Bi M line at 2.419 keV and the Rh L_*α*1_ line at 2.697 keV, are rather close, which makes it difficult to separate them in the measurement. This causes an inherent error of about 1-2 at.% in the compositions derived from WDX/EDX measurements.

Differential thermal analyses (DTA) were performed on a DSC 404F1 Pegasus (Netzsch, Selb, Germany), applying evacuated silica glass crucibles (~ 10^−3^ mbar). The temperature program included two heating/cooling cycles at a rate of 5 K/min starting from 200 °C up to about 30° above the estimated liquidus temperature, not exceeding a maximum temperature of 1040 °C. Two samples with 23 and 30 at.% Rh, respectively, were measured only up to 520 °C. Temperature measurements were performed with type S (Pt/PtRh) thermocouples, calibrated at the melting points of Ag, Au, Bi, Sb, and Sn. Zr was used as reference material.

Invariant effects were evaluated from the peak onset, both on heating and cooling, liquidus effects were evaluated from the peak maximum on heating and from the peak onset on cooling. Generally, the effects evaluated in the first heating run of annealed samples were deemed most reliable.

## Results and Discussion

**Fig. 1:**
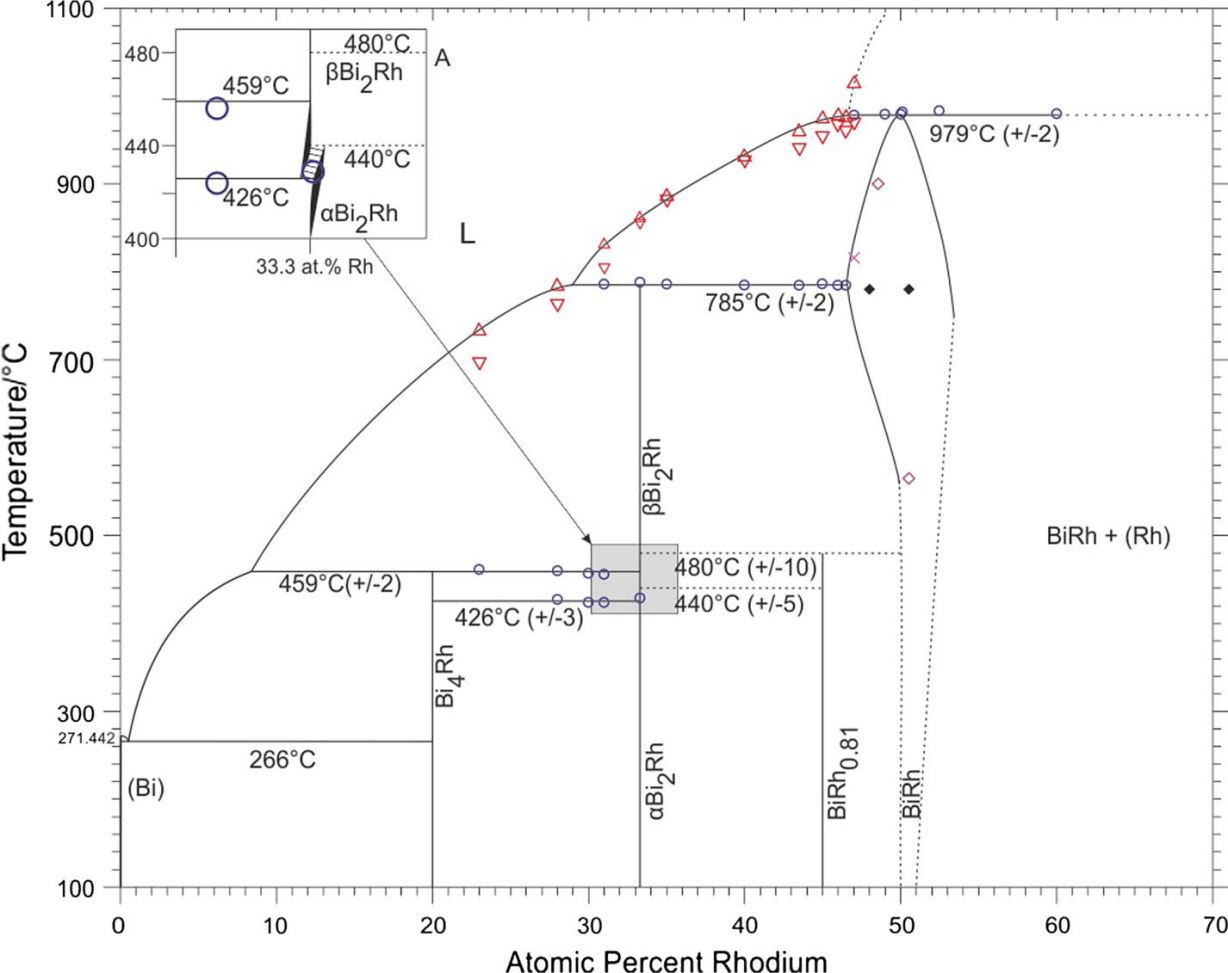
Bi-Rh phase diagram according to the present results without the metastable Bi_3_Rh phase; dashed lines are estimated. The Bi-rich part was taken from Okamoto.[26] Circle, invariant thermal effects; triangle, liquidus on heating; inverted triangle, liquidus on cooling; cross, thermal effects related to phase boundaries; open diamond, phase boundary extrapolated from lattice parameter *a* of BiRh phase as a function of Rh concentration (Fig. 7); filled diamond, values from Ross and Hume-Rothery.[9] The insert A shows a detail of the *α*-Bi_2_Rh $\rightleftharpoons$ *β*-Bi_2_Rh region at 33.3 at.% Rh in the case that metastable Bi_3_Rh is not present

**Fig. 2:**
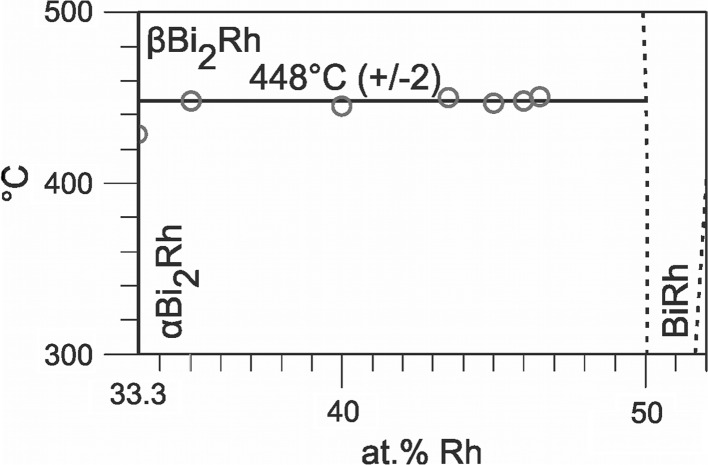
Detail of Bi-Rh phase diagram between 33.3 and 52 at.% Rh and 300-500 °C, without BiRh_0.81_; dashed lines are estimated. Circle: invariant thermal effects. The 448 ± 2 °C is the transition temperature of *α*-Bi_2_Rh $\rightleftharpoons$ *β*-Bi_2_Rh if BiRh_0.81_ is not present. All thermal effects are listed in Table 4

**Fig. 3:**
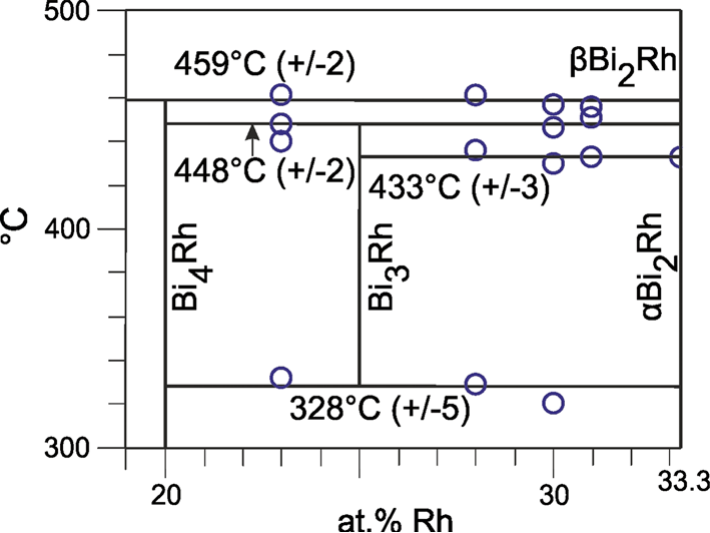
Detail of Bi-Rh phase diagram between 19 and 33.3 at.% Rh and 300-500 °C. Circle, invariant thermal effects. If the metastable Bi_3_Rh is present the transition temperature of *α*-Bi_2_Rh $\rightleftharpoons$ *β*-Bi_2_Rh increases by a few degrees from 426 ± 3 to 433 ± 3 °C. All thermal effects are listed in Table 4

**Fig. 4:**
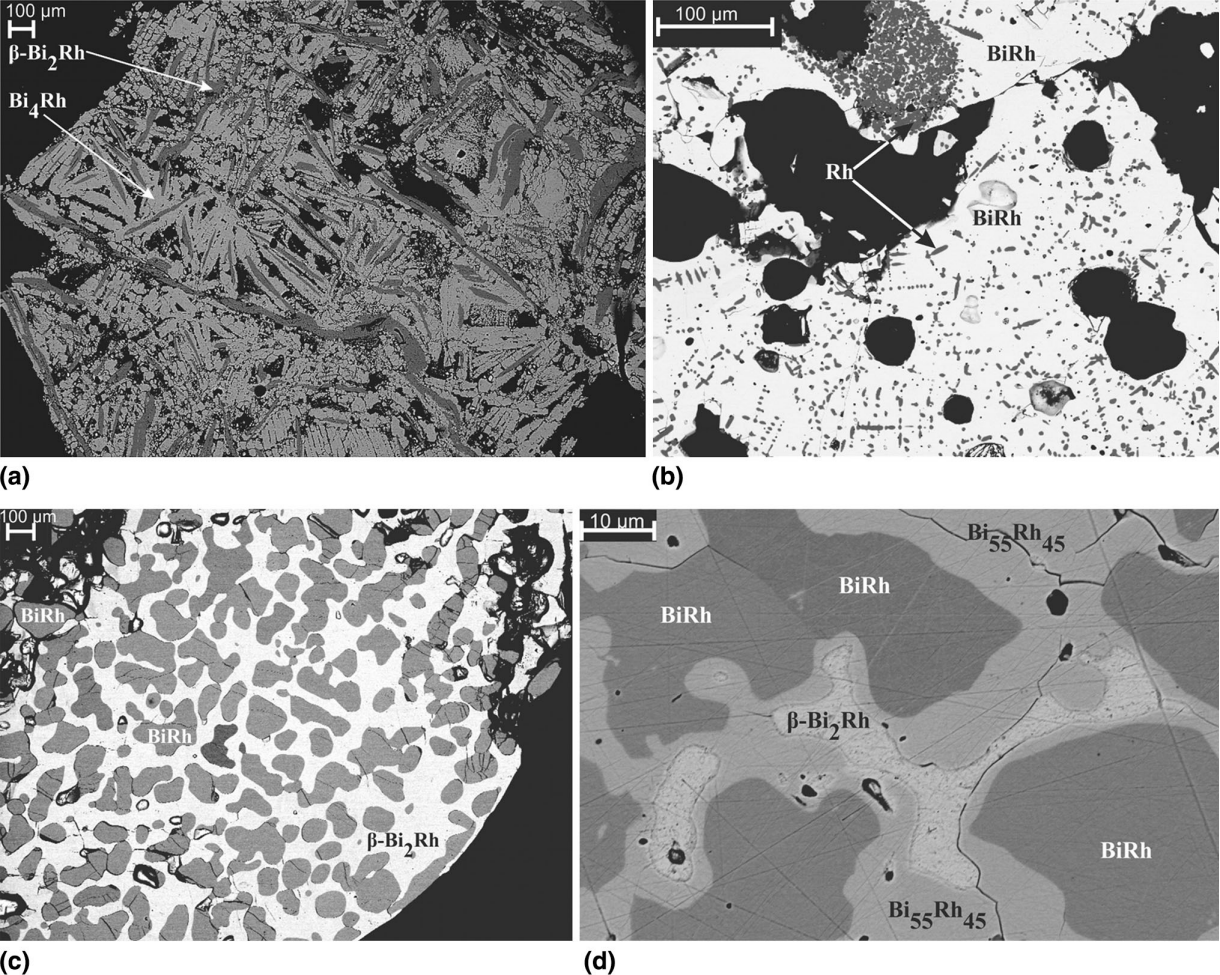
SEM backscattered electron images of selected samples (black structures are either holes or cracks caused by sample preparation). (a) Bi_71.6_Rh_28.4_ annealed at 430 °C, shows *β*-Bi_2_Rh phase surrounded by Bi_4_Rh matrix. (b) Bi_43.8_Rh_56.2_ annealed at 750 °C, shows small spots of Rh and BiRh phase. (c) Bi_57.5_Rh_42.5_ annealed at 750 °C, shows BiRh and *β*-Bi_2_Rh phases. (d) Bi_55_Rh_45_ annealed at 440 °C shows BiRh, *β*-Bi_2_Rh, BiRh_0.81_ (Bi_55_Rh_45_) phases

 Sample compositions, heat treatments and results of powder-XRD and EDX/WDX are summarized in Table 3. Table 4 contains the results of DTA measurements. A partial Bi-Rh phase diagram for compositions up to 70 at.% Rh based on data obtained in this study and data from Ref 9 and 26, is presented in Fig. 1. Figure 2 and 3 show detail sections for the composition ranges 33.3-52 at.% Rh (if BiRh_0.81_ is absent) and 19-33.3 at.% Rh (if metastable BiRh_3_ is present). The Bi-rich part between 0 and 20 at.% Rh was entirely taken from Okamoto.[26] Figure 4(a), (b), (c), and (d) show SEM backscattered images for selected samples.

**Table 3:** Experimental phase compositions and cell parameters of selected samples

Sample comp., at.%	Annealing duration/ *T* d/°C	XRD			*WDX*/EDX(e)	
		Phase	Lattice parameters		Rh, at.%	Bi, at.%
			Å	deg		
Bi_77_Rh_23_	28/430	*β*-Bi_2_Rh	*a* = 6.737(6) *b* = 7.027(1) *c* = 7.058(1)	*α* = 104.78 *β* = 100.72 *γ* = 105.75	34.6	65.4
		Bi_4_Rh	*a* = 14.930(5)		21.0	79.0
	14/300	*α*-Bi_2_Rh	*a* = 6.919(4) *b* = 6.794(3) *c* = 6.961(5)	*β* = 117.73(5)	…	…
		Bi_4_Rh	*a* = 14.930(6)		…	…
Bi_72_Rh_28_	10/730	Bi(a)	*a* = 4.547(3) *c* = 11.866(2)		0.0	100.0
		*β*-Bi_2_Rh	*a* = 6.739(3) *b* = 7.025(7) *c* = 7.057(7) *α* = 104.82	*β* = 100.72 *γ* = 105.77	35.0	65.0
	14/400	*α*-Bi_2_Rh	*a* = 6.920(1) *b* = 6.794(1) *c* = 6.963(2)	*β* = 117.73(1)	…	…
		Bi_4_Rh	*a* = 14.931(1)		…	…
Bi_71.6_Rh_28.4_	28/430	*β*-Bi_2_Rh	*a* = 6.738(3) *b* = 7.021(4) *c* = 7.058(4)	*α* = 104.76 *β* = 100.77 *γ* = 105.75	34.7	65.3
		Bi_4_Rh	*a* = 14.931(5)		21.2	78.8
Bi_70_Rh_30_	14/750	Bi	*a* = 4.546(4) *c* = 11.862(2)		0.0	100.0
		*β*-Bi_2_Rh	*a* = 6.738(3) *b* = 7.026(8) *c* = 7.061(8)	*α* = 105.06 *β* = 100.64 *γ* = 105.74	35.1	64.9
Bi_69_Rh_31_	10/730	Bi(a)	*a* = 4.547(4) *c* = 11.864(2)		0.0	100.0
		*β*-Bi_2_Rh	*a* = 6.738(3) *b* = 7.024(7) *c* = 7.060(8)	*α* = 104.81 *β* = 100.70 *γ* = 105.77	34.8	65.2
Bi_66.7_Rh_33.3_	46/400	*α*-Bi_2_Rh	*a* = 6.920(2) *b* = 6.794(2) *c* = 6.962(2)	*β* = 117.73(2)	35.3	64.7
Bi_65_Rh_35_	10/730	*β*-Bi_2_Rh	*a* = 6.737(3) *b* = 7.023(6) *c* = 7.057(6)	*α* = 104.81 *β* = 100.73 *γ* = 105.77	34.9	65.1
		BiRh	*a* = 4.079(3) *c* = 5.671(6)		49.4	50.6
Bi_60_Rh_40_	10/730	*β*-Bi_2_Rh	*a* = 6.737(2) *b* = 7.024(2) *c* = 7.057(3)	*α* = 104.78 *β* = 100.74 *γ* = 105.77	35.1	64.9
		BiRh	*a* = 4.079(6) *c* = 5.671(1)		49.2	50.8
Bi_57.5_Rh_42.5_	16/750	*β*-Bi_2_Rh	*a* = 6.738(4) *b* = 7.024(5) *c* = 7.057(3)	*α* = 104.73 *β* = 100.76 *γ* = 105.75	*34.7*	*65.3*
		BiRh	*a* = 4.077(4) *c* = 5.673(7)		*48.8*	*51.2*
Bi_56.5_Rh_43.5_	16/750	*β*-Bi_2_Rh	*a* = 6.737(1) *b* = 7.024(1) *c* = 7.059(1)	*α* = 104.76 *β* = 100.77 *γ* = 105.77	35.0	65.0
		BiRh	*a* = 4.076(3) *c* = 5.673(5)		49.9	50.1
Bi_55_Rh_45_	16/750	*β*-Bi_2_Rh	*a* = 6.738(5) *b* = 7.024(4) *c* = 7.057(4)	*α* = 104.76 *β* = 100.75 *γ* = 105.77	*35.2*	*64.8*
		BiRh	*a* = 4.077(1) *c* = 5.673(4)		*49.2*	*50.8*
	7/438(b)	*α*-Bi_2_Rh	*a* = 6.919(4) *b* = 6.796(3) *c* = 6.960(4)	*β* = 117.70(4)	…	…
		BiRh	*a* = 4.091(4) *c* = 5.668(9)		…	…
	17/438(b)	*β*-Bi_2_Rh	*a* = 6.737(6) *b* = 7.027(2) *c* = 7.053(2)	*α* = 104.84 *β* = 100.73 *γ* = 105.77	35.0	65.0
		BiRh	*a* = 4.090(1) *c* = 5.668(2)		50.6	49.4
		BiRh_0.81_	*a* = 6.174(5) *b* = 3.783(3) *c* = 6.550(5)		44.8	55.2
Bi_55_Rh_45_	27/440	*β*-Bi_2_Rh	*a* = 6.737(8) *b* = 7.019(9) *c* = 7.057(6)	*α* = 104.70 *β* = 100.75 *γ* = 105.81	35.2	64.8
		*α*-Bi_2_Rh	*a* = 6.925(2) *b* = 6.793(1) *c* = 6.953(2)	*β* = 117.82(2)		
		BiRh	*a* = 4.091(4) *c* = 5.667(5)		50.5	49.5
		BiRh_0.81_	*a* = 6.175(2) *b* = 3.782(4) *c* = 6.550(7)		44.7	55.3
Bi_54_Rh_46_	16/750	*β*-Bi_2_Rh	*a* = 6.737(8) *b* = 7.024(8) *c* = 7.056(2)	*α* = 104.71 *β* = 100.74 *γ* = 105.78	*34.3*	*65.7*
		BiRh	*a* = 4.077(1) *c* = 5.673(4)		*48.6*	*51.4*
Bi_53.5_Rh_46.5_	16/565	*β*-Bi_2_Rh	*a* = 6.737(3) *b* = 7.024(3) *c* = 7.058(3)	*α* = 104.77 *β* = 100.75 *γ* = 105.77	35.0	65.0
		BiRh	*a* = 4.0876(3) *c* = 5.6685(6)		48.9	51.1
	7/900(c)	*β*-Bi_2_Rh	*a* = 6.735(2) *b* = 7.021(2) *c* = 7.059(2)	*α* = 104.78 *β* = 100.74 *γ* = 105.74	34.8	65.2
		BiRh	*a* = 4.082(6) *c* = 5.671(6)		49.4	50.6
Bi_53_Rh_47_	16/750	BiRh	*a* = 4.078(4) *c* = 5.672(7)		*49.4*	*50.6*
Bi_52.3_Rh_47.7_	7/950(c)	*β*-Bi_2_Rh	*a* = 6.736(3) *b* = 7.037(3) *c* = 7.064(3)	*α* = 104.71 *β* = 100.80 *γ* = 105.8	35.1	64.9
		BiRh	*a* = 4.089(3) *c* = 5.668(5)		50.4	49.6
Bi_52_Rh_48_	16/750	BiRh	*a* = 4.080(3) *c* = 5.672(4)		*49.0*	*51.0*
Bi_51_Rh_49_	16/750	BiRh	*a* = 4.084(4) *c* = 5.670(5)		*49.7*	*50.3*
Bi_50_Rh_50_	16/750	BiRh	*a* = 4.087(6) *c* = 5.669(8)		*49.8*	*50.2*
Bi_50_Rh_50_	21/438(d)	BiRh	*a* = 4.092(2) *c* = 5.667(5)		50.9	49.1
		BiRh_0.81_	*a* = 6.175(2) *b* = 3.781(3) *c* = 6.549(3)		45.1	54.9
Bi_47.5_Rh_52.5_	16/750	BiRh	*a* = 4.092(2) *c* = 5.668(1)		*50.0*	*50.0*
Bi_43.8_Rh_56.2_	14/750	Rh	*a* = 3.8045(5)		99.8	0.2
		BiRh	*a* = 4.097(3) *c* = 5.667(6)		51.1	48.9
Bi_40_Rh_60_	14/750	Rh	*a* = 3.8047(9)		99.4	0.6
		BiRh	*a* = 4.097(3) *c* = 5.667(5)		50.7	49.3

(a) Precipitated out of the liquid on quenching; (b) re-annealed at 438 °C; (c) *β*-Bi_2_Rh segregated during quenching; (d) negligible quantities of *β*-Bi_2_Rh and unreacted Rh detected by XRD; (e) compositions in italics from *WDX*

**Table 4:** Summary of measured thermal effects

Sample comp., at.%	Annealing duration, *T* d/°C	Thermal analysis			
		Heating, °C			Cooling, °C
		Invariant effects	Other effects	Liquidus	Liquidus
Bi_77_Rh_23_	28/430	*331*; *448*; 461	…	734	697
Bi_72_Rh_28_	10/730	*329*; *436*; *453*; 427; 460	…	785	763
Bi_70_Rh_30_	14/750	*321*; *430*; *447*; 423; 457	…	…	…
Bi_69_Rh_31_	10/730	*433*; *451*; 423; 456; 785	…	832	805
Bi_66.7_Rh_33.3_	46/400	*433*; 429; 784	…	863	855
Bi_65_Rh_35_	10/730	448(a); 786	…	888	881
Bi_60_Rh_40_	10/730	445(a); 785	…	932	927
Bi_56.5_Rh_43.5_	14/750	450(a); 785	…	961	941
Bi_55_Rh_45_	15/750	446(a); 785	…	975	954
Bi_54_Rh_46_	15/750	448(a); 785	…	979	966
Bi_53.5_Rh_46.5_	14/565	451(a); 785	…	978	961
Bi_53_Rh_47_	15/750	979	817	1016	970
Bi_51_Rh_49_	15/750	977	…	…	…
Bi_50_Rh_50_	23/438	978	…	…	…
Bi_47.5_Rh_52.5_	15/750	979	…	…	…
Bi_40_Rh_60_	14/750	980	…	…	…

Italics: second heating; (a) metastable, only first heating, not appearing in second heating loop

### Liquidus Curve

The liquidus curve in Fig. 1 is based on DTA results of the quenched samples. Its shape, together with the corresponding invariant effects, indicates that the phases BiRh and *β*-Bi_2_Rh are formed by peritectic reactions, i.e., liquid with about 47 at.% Rh reacts at 979 ± 2 °C with Rh to form BiRh, and liquid with about 29 at.% Rh reacts at 785 ± 2 °C with BiRh to form *β*-Bi_2_Rh. These values are in good agreement with results of Ross and Hume-Rothery[9] (46.0 at.% Rh at 977 °C for BiRh and 29.5 at.% Rh at 780 °C for Bi_2_Rh), based on results from cooling curves.

The shape of the liquidus curve at higher temperatures above the peritectic decomposition of BiRh could not be determined, because no signals were found except in the sample Bi_53_Rh_47_ (1016 °C). This indicates a very steep ascent to the melting point of Rh.

### Composition Range 20-33.3 at.% Rh

The obtained results differ somewhat from the phase diagram assessed by Okamoto.[26] In particular, no hint was found for the existence of the Bi_3_Rh phase in the XRD or EDX measurements of annealed samples. All samples in the investigated composition range showed exclusively Bi_4_Rh and *α*- or *β*-Bi_2_Rh, independently of their annealing temperatures (Table 3).

DTA analyses of these samples show on first heating an extrapolated onset of the endothermic effect at 460 ± 3 °C which represents the peritectic reaction (L + *β*-Bi_2_Rh $\rightleftharpoons$ Bi_4_Rh) temperature (Table 4). This value is slightly lower than the one reported by Weitzer et al.[25] (466 °C) but in perfect agreement with the 460 °C assessed by Predel.[24] Two thermal effects with average temperatures of 328 ± 5 and 448 ± 2 °C were observed only in the corresponding second heating runs.

It was concluded that these correspond to the eutectoid and peritectoid reaction temperatures of (metastable) Bi_3_Rh, described by Weitzer et al.[25] into *α*-Bi_2_Rh and Bi_4_Rh at 336 °C (Bi_3_Rh $\rightleftharpoons$ Bi_4_Rh + *α*-Bi_2_Rh) and 433 °C (Bi_4_Rh + β-Bi_2_Rh $\rightleftharpoons$ Bi_3_Rh). XRD measurements of samples after the DTA analyses did not show the Bi_3_Rh phase.

Combining the results of all analyses (Tables 3 and 4) suggests that Bi_3_Rh is actually a metastable phase which does not form in annealed samples, fully consistent with observations by Ross and Hume-Rothery[9] who were likewise not able to detect the Bi_3_Rh phase. It is probably also in line with the report by Weitzer et al.[25] who considered Bi_3_Rh to be a stable compound but found that it could only be obtained by annealing a mixture of Bi_2_Rh with a metastable phase, i.e., Bi_14_Rh_3_.

### The Phase Bi_2_Rh

WDX/EDX measurements of all samples containing *β*-Bi_2_Rh show an average composition of 34.9 ± 0.5 at.% Rh independent of the annealing temperature. This value differs somewhat from the stoichiometric composition of 33.3 at.% Rh. It is thought that this discrepancy is due to problems with the WDX/EDX measurements: the characteristic XRD radiation spectra of Bi and Rh, especially the Bi M line at 2.419 keV and the Rh L_*α*1_ line at 2.697 keV, are rather close, which makes it difficult to separate them in the measurement (see section 3.2). DTA results of samples containing the Bi_2_Rh phase show an invariant reaction at 785 ± 2 °C which presents the peritectic reaction (L + BiRh $\rightleftharpoons$ *β*-Bi_2_Rh). This temperature is in better agreement with the results by Ross and Hume-Rothery[9] (780 °C) and Kjekshus and Rakke[29] (778 ± 4 °C) than with those reported by Ref 16, 18, and 22 where it is somewhat lower.

Weitzer et al.[25] indicated the transition of *α*- into *β*-Bi_2_Rh at 370 °C which could not be verified here, neither by DTA nor by powder XRD measurements of annealed samples. The data obtained in this study indicate a much more complex transition, its temperature being different below and above 33.3 at.% Rh, respectively, and also depending on the presence or absence of metastable Bi_3_Rh or stable BiRh_0.81_ phase.

**Fig. 5:**
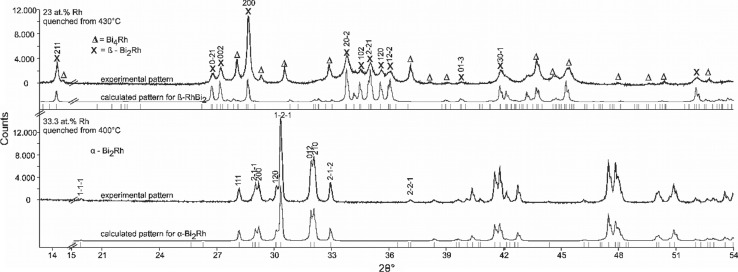
Powder XRD pattern of a sample with 23 at.% Rh quenched from 430 °C (top), and 33.3 at.% Rh quenched from 400 °C (bottom). The experimental pattern of the sample (above) and the calculated contribution of *α*-, and *β*-Bi_2_Rh (below) are shown for both forms. The Miller indices are given for the ten first occurring reflexes of Bi_2_Rh based on the Bi_2_Rh structure given by Ref 31 and 40. Both peaks at 29.8 and 40.0 2*θ*° in the bottom pattern are not corresponding to any phase. Excluded range between 15 and 20 2*θ*° does not show any reflexes

**Fig. 6:**
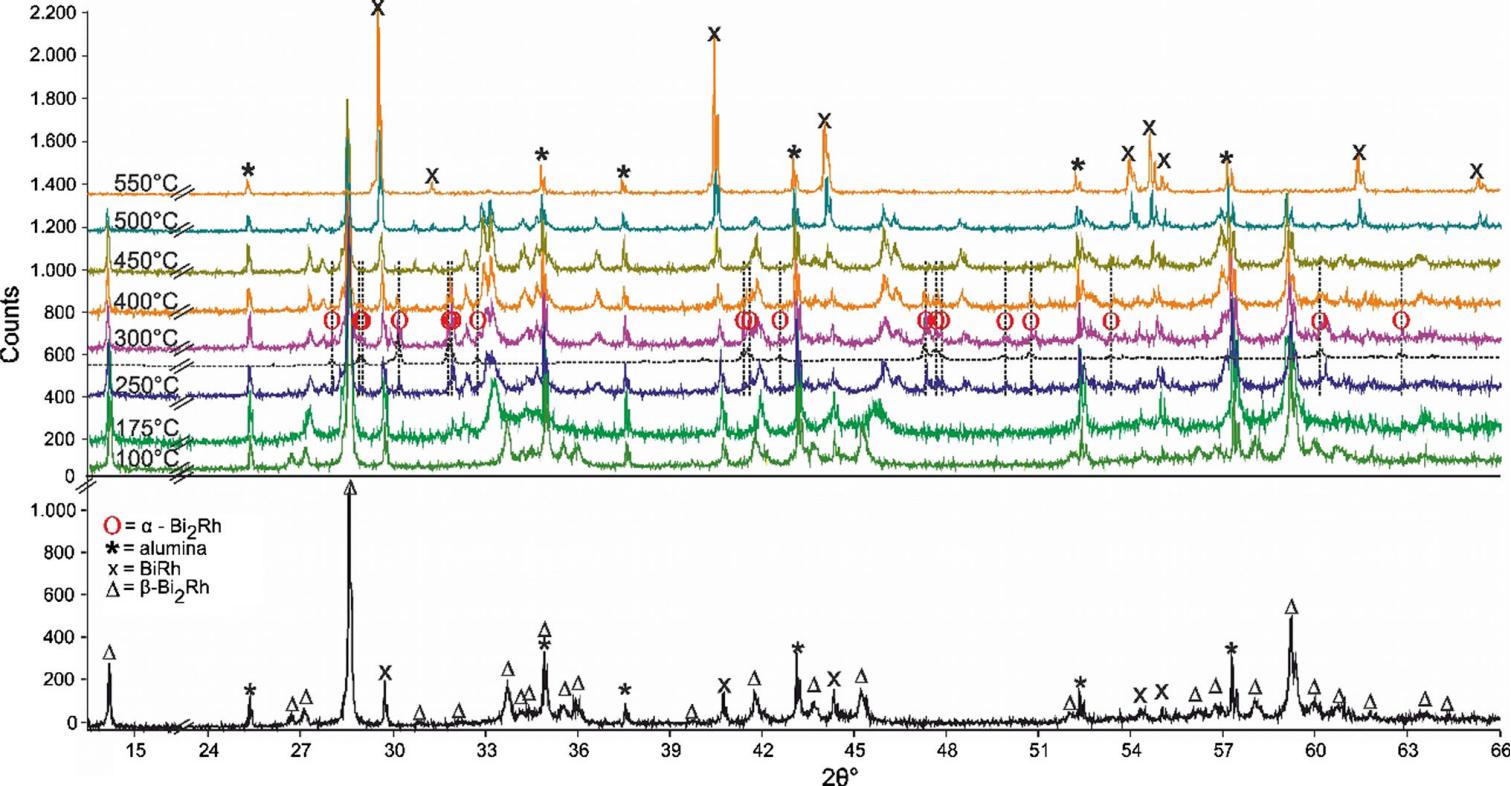
Powder XRD pattern of a sample with 35 at.% Rh quenched from 730 °C measured at ambient conditions (bottom) and at different temperatures (top). The dashed line between the 250 and 300 °C patterns shows the calculated XRD reflexes of *α*-Bi_2_Rh at 300 °C, marked with ϴ. For better illustration these reflexes are marked with vertical lines. The refinement is based on the structures given by Ref 9, 31, and 40. Peaks marked with * correspond to alumina sample carrier. Excluded range between 15 and 24 2*θ*° does not show any reflexes

XRD analysis of a sample with the stoichiometric composition Bi_66.7_Rh_33.3_, annealed for 46 days at 400 °C, shows only *α*-Bi_2_Rh (Fig. 5, bottom). A sample with 35 at.% Rh, quenched from 730 °C, showed originally *β*-Bi_2_Rh and BiRh (Fig. 6, bottom); this sample was then subjected to high temperature XRD. On heating, the phase *α*-Bi_2_Rh appears at 250 °C and disappears between 400 and 450 °C indicating a transition *α*-Bi_2_Rh $\rightleftharpoons$ *β*-Bi_2_Rh below 450 °C on the Rh-rich side. Unfortunately, Bi_2_Rh starts to decompose above 500 °C due to the evaporation of Bi in the evacuated high-temperature x-ray chamber and disappears completely around 550 °C, leaving only the BiRh phase.

The obtained XRD results are in good agreement with DTA data, which show in all samples with Rh contents ≥ 35 at.%, independently of the annealing parameters, an invariant effect at 448 ± 2 °C (Table 4) if the new phase BiRh_0.81_ is not present. Quenching experiments indicate that this transition temperature is approximately 440 ± 5 °C if the BiRh_0.81_ phase is present (Table 3).

The *α* $\rightleftharpoons$ *β* transition temperature in the composition range below 33.3 at.% Rh is 426 ± 3 or 433 ± 3 °C, depending on the absence or presence of the metastable Bi_3_Rh phase. XRD investigations of samples with 23 (Fig. 5, top) and 28.4 at.% Rh, annealed at 430 °C, (Table 3) reveal the *β* modification of Bi_2_Rh, the sample with 28 at.% Rh, annealed at 400 °C, showed the *α* modification. These XRD results are in good agreement with DTA data, which show for all samples, independent of the annealing temperature, thermal arrests between 423 and 427 °C (Table 4). They also agree with values of Kuz´min and Zhuravlev[21] (420 °C, DTA), Ross and Hume-Rothery[9,23] (430, 425 °C, high temperature XRD), Kjekshus and Rakke[29] (425 ± 3 °C, DTA and quenching experiments) and Fjellvag and Furuseth[30] (427 ± 10 °C, high temperature XRD).

It is concluded that this difference in the *α* $\rightleftharpoons$ *β* transition temperature for compositions below and above 33.3 at.% Rh is caused by a eutectoid on the Bi-rich side (*β*-Bi_2_Rh $\rightleftharpoons$ Bi_4_Rh + *α*-Bi_2_Rh) and a peritectoid on the Rh-rich side (*α*-Bi_2_Rh $\rightleftharpoons$ *β*-Bi_2_Rh + BiRh/BiRh_0.81_) as indicated in the inset in Fig. 1.

If the metastable Bi_3_Rh phase is present, DTA measurements of samples between 28 and 31 at.% Rh show, independent of the annealing parameters, an invariant effect at 433 ± 3 °C for the *α* $\rightleftharpoons$ *β* transition (Table 4). But once again it must be pointed out that metastable Bi_3_Rh only occurs in second heating loops. A graphical presentation of the reported values is given in Fig. 1 to 3. The average transition temperature of 433 ± 3 °C (< 33.3 at.% Rh) corresponds to the value of 433 °C, which Weitzer et al.[25] reported for the peritectoid decomposition of the Bi_3_Rh phase.

### The Phase BiRh

The obtained homogeneity range of the phase BiRh differs from data reported in the literature[9,17] as well as does the temperature of the peritectic.[16,20,26]

**Fig. 7:**
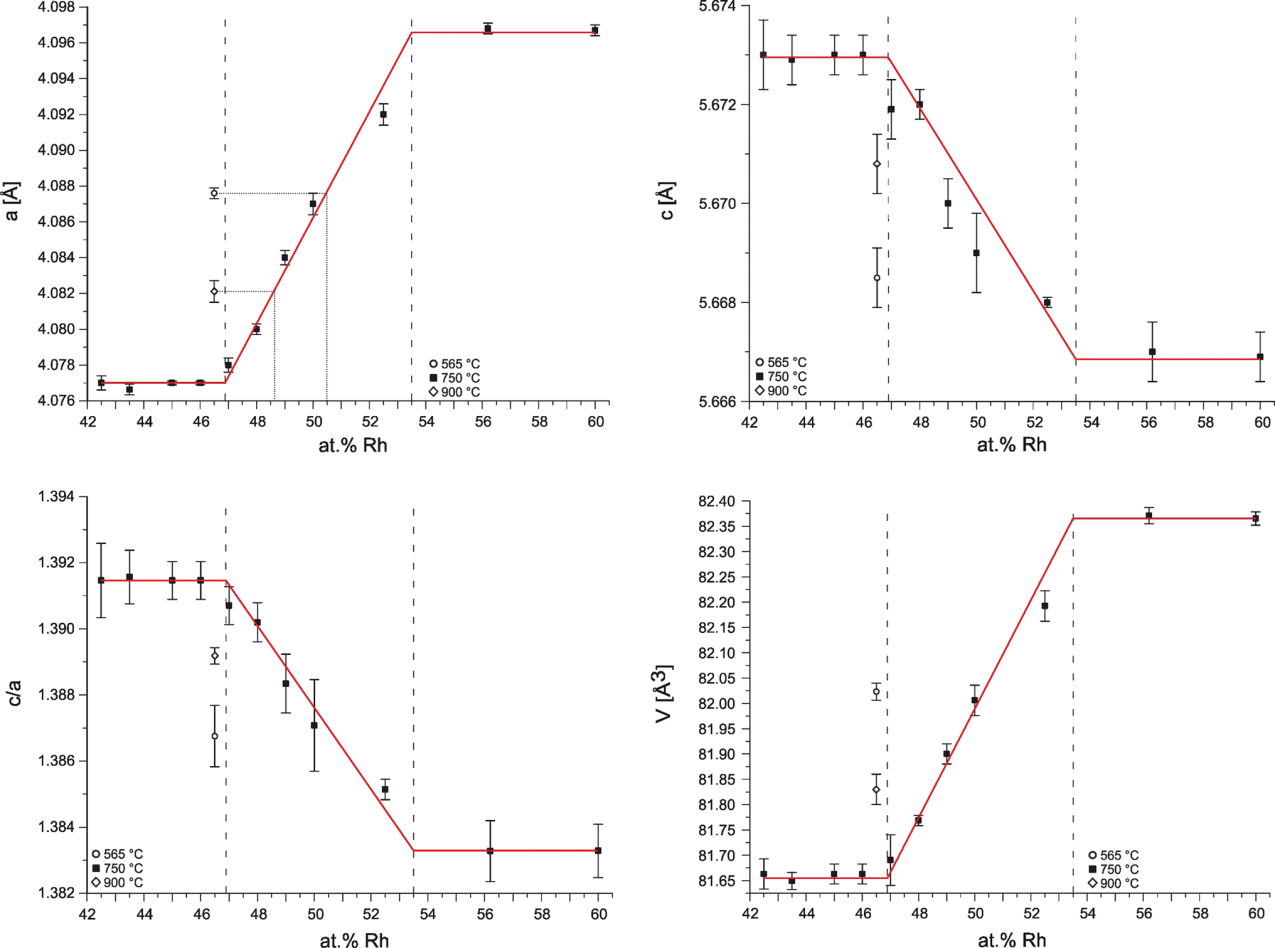
Lattice parameters *a*, *c*, *c/a*, *V* of BiRh as a function of Rh concentration. The dashed lines indicate the proposed phase boundaries at 750 °C

In particular, DTA results of samples between 47 and 60 at.% Rh show an invariant reaction at 979 ± 2 °C which is the peritectic decomposition temperature of BiRh. This value conforms to the 977 °C reported by Ross and Hume-Rothery[9] but is 20 °C lower than assessed by Okamoto.[26] Results on the homogeneity range of BiRh are somewhat contradictory: WDX/EDX results of samples in the adjacent two-phase fields, annealed at 750 °C seem to indicate a rather narrow stability range for BiRh between about 49.8 and 50.9 at.% Rh. These values are in contrast to powder XRD investigations of samples between 47 and 52.5 at.% Rh which show only BiRh and no second phase. As described in section 4.3, the characteristic XRD radiation spectra of Bi and Rh are rather close, which makes it difficult to separate them in the measurement. Therefore, the EDX/WDX values may show unusually high errors and the homogeneity range of BiRh, as it is shown in Fig. 1, is deduced from the lattice parameter measurements (Table 3 and Fig. 7).

The present results are in significant contrast to the values reported in the literature, particularly in Ref 16, 17, 24, and 26. Of these, Glagoleva and Zhdanov[17] annealed their samples at 800 °C but let them slowly cool down to room temperature instead of quenching; thus, their lattice parameters correspond obviously to some lower temperature. The results can be best compared to data by Ross and Hume-Rothery[9] who determined the homogeneity range of BiRh at 780 °C as 48.0-50.6 at.% Rh; this is in reasonable agreement with the present phase boundary on the Bi-rich side at 750 °C, i.e., 46.9 at.% Rh, deduced from lattice parameter investigations. Extrapolating the lattice parameter *a* of a sample with 46.5 at.% Rh (annealed at different temperatures) to the plotted regression line (Fig. 7) indicates a pronounced retrograde solubility of BiRh, resulting in a phase boundary of about 48 at.% Rh at 900 and about 50.5 at.% Rh at 565 °C. On the Rh-rich side, the lattice parameter measurements give a phase boundary of 53.5 at.% Rh at 750 °C which points to a much wider homogeneity range of BiMn than reported in Ref 9

### The Phase BiRh_0.81_

**Fig. 8:**
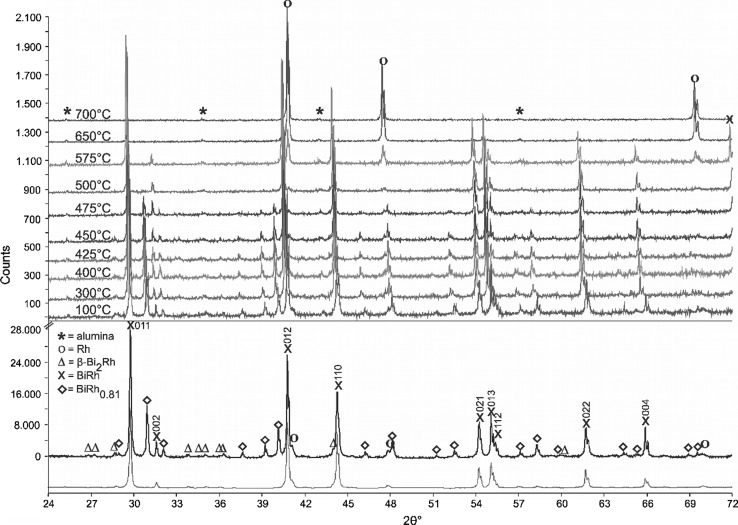
Powder XRD patterns of a sample with 50 at.% Rh quenched from 438 °C measured at ambient conditions (bottom), and at different temperatures (top). The experimental pattern of the sample (above) and the calculated contribution of Rh, *β*-Bi_2_Rh, BiRh, BiRh_0.81_ (below) are shown. The Miller indices are given for the BiRh reflexes. The refinement is based on the structures given by Ref 9, 31, and 44. Peaks marked with * correspond to the alumina sample carrier

SEM–EDX investigations of a sample with 50 at.% Rh, annealed for 21 days at 438 °C, showed, besides the known phases BiRh and Bi_2_Rh, also a phase with a composition of Bi_55_Rh_45_ (54.9 at.% Bi and 45.1 at.% Rh). High temperature XRD measurements of the same sample (Table 5 and Fig. 8) showed that the reflexes of this phase disappear between 475 and 500 °C.

**Table 5:** Lattice parameters of Bi_50_Rh_50_ as a function of temperature, quenched from 438 °C

*T*, °C	Lattice parameters								
	BiRh_0.81_					BiRh			
	*a*, Å	*b*, Å	*c*, Å	*V*, Å^3^	*c*/*b*	*a*, Å	*c*, Å	*V*, Å^3^	*c*/*a*
25	6.175(3)	3.781(4)	6.549(5)	152.94(2)	1.732(1)	4.092(5)	5.667(1)	82.21(3)	1.384(2)
50	6.179(3)	3.784(4)	6.552(5)	153.19(2)	1.732(1)	4.093(8)	5.670(2)	82.30(4)	1.385(3)
75	6.182(3)	3.785(4)	6.555(5)	153.39(2)	1.732(1)	4.095(7)	5.673(1)	82.40(4)	1.385(2)
100	6.185(3)	3.786(4)	6.557(5)	153.58(2)	1.732(1)	4.096(6)	5.675(1)	82.49(3)	1.385(2)
125	6.187(3)	3.787(5)	6.559(5)	153.74(2)	1.732(2)	4.097(7)	5.678(1)	82.57(3)	1.386(2)
150	6.190(2)	3.788(4)	6.561(5)	153.89(2)	1.732(1)	4.098(7)	5.681(1)	82.65(3)	1.386(1)
175	6.192(2)	3.789(4)	6.563(5)	154.02(2)	1.732(1)	4.099(7)	5.683(1)	82.71(3)	1.386(2)
200	6.194(2)	3.790(4)	6.564(5)	154.14(2)	1.732(1)	4.100(6)	5.685(1)	82.76(3)	1.386(2)
225	6.196(2)	3.791(4)	6.566(5)	154.27(2)	1.732(1)	4.100(6)	5.686(1)	82.82(3)	1.387(2)
250	6.198(2)	3.792(4)	6.568(5)	154.40(2)	1.732(1)	4.101(6)	5.689(1)	82.88(3)	1.387(1)
275	6.200(2)	3.793(3)	6.569(5)	154.53(2)	1.731(1)	4.102(6)	5.691(1)	82.94(3)	1.387(2)
300	6.203(2)	3.794(4)	6.570(5)	154.67(2)	1.732(1)	4.103(6)	5.693(1)	83.01(3)	1.387(2)
325	6.205(2)	3.795(4)	6.573(5)	154.83(2)	1.732(1)	4.104(6)	5.695(1)	83.08(3)	1.388(2)
350	6.207(2)	3.796(3)	6.575(4)	154.95(1)	1.732(1)	4.105(6)	5.697(1)	83.14(3)	1.388(2)
375	6.209(2)	3.797(3)	6.578(3)	155.11(2)	1.733(1)	4.106(6)	5.699(1)	83.22(3)	1.388(2)
400	6.212(2)	3.799(2)	6.579(3)	155.30(1)	1.733(1)	4.107(5)	5.702(9)	83.29(2)	1.388(1)
425	6.214(2)	3.800(3)	6.582(4)	155.47(1)	1.732(1)	4.107(5)	5.705(9)	83.37(2)	1.389(1)
450	6.217(2)	3.802(2)	6.585(3)	155.68(2)	1.732(1)	4.109(4)	5.708(8)	83.45(2)	1.389(1)
475	6.220(5)	3.804(7)	6.587(9)	155.89(4)	1.731(2)	4.110(3)	5.710(7)	83.54(2)	1.389(1)
500	–	–	–	–	–	4.114(3)	5.712(5)	83.73(1)	1.388(1)
525	–	–	–	–	–	4.118(3)	5.713(5)	83.91(1)	1.387(1)
550	–	–	–	–	–	4.120(2)	5.715(3)	84.02(7)	1.387(1)
575	–	–	–	–	–	4.121(2)	5.717(4)	84.09(1)	1.387(1)
600	–	–	–	–	–	4.121(2)	5.720(3)	84.17(9)	1.388(1)
625	–	–	–	–	–	4.122(4)	5.723(8)	84.23(2)	1.388(1)
650	–	–	–	–	–	4.123(7)	5.726(2)	84.31(4)	1.389(3)

**Fig. 9:**
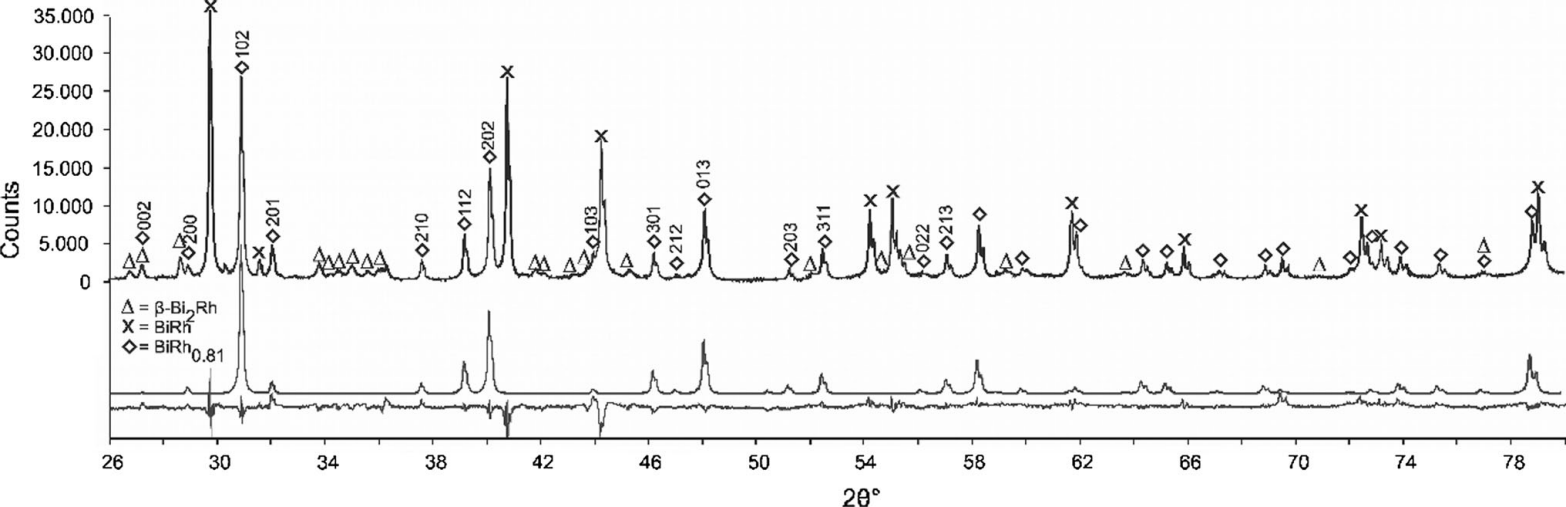
Powder XRD pattern of a sample with 45 at.% Rh quenched from 440 °C measured at ambient conditions. The pattern contains BiRh_0.81_ together with the phases BiRh and *β*-Bi_2_Rh as well as traces of *α*-Bi_2_Rh. The experimental pattern (above), the calculated pattern of BiRh_0.81_ (middle) and the difference curve (bottom) are shown. The Miller indices are given for the first 15 BiRh_0.81_ reflexes. The refinement is based on the structures given by Ref 9 and 31. One significant peak at 2*θ* = 30.26° and some other peaks not marked correspond to *α*-Bi_2_Rh. The *α*-Bi_2_Rh phase is present with < 1 wt.% in the sample

Indexing of the unknown diffraction lines and subsequent refinement of the powder XRD pattern of a sample with the composition Bi_55_Rh_45_, annealed for 27 days at 440 °C (Table 3), indicates that the new phase is orthorhombic (*Pnma*) with the lattice parameters *a* = 6.1753(9) Å, *b* = 3.7817(1) Å, *c* = 6.5506(1) Å. Refinement was successful using the structural model for the MnP-type structure, which is closely related to the NiAs type of BiRh. The refined occupancy factor for Rh is 0.81(3), in excellent agreement with the phase composition of Bi_55.3_Rh_44.7_ determined by SEM–EDX. Therefore, this new phase was designated BiRh_0.81_. The refined atomic coordinates and structural parameters are given in Table 6 and Fig. 9 displays a 2*θ* segment of the powder XRD pattern between 26° and 80°. Detailed structural information was deposited with *Fachinformationszentrum Karlsruhe* 1 and can be obtained on quoting the depository number CSD 433459.

**Table 6:** Structural parameters for BiRh_0.81_, quenched from 440 °C

Atoms	Site	Atomic coordinate (*x*, *y*, *z*)	SOF	*U*_ISO_
Rh	4	− 0.0035(6), 0.25, 0.1944(5)	0.81(3)	0.98(2)
Bi	4	0.2019(2), 0.25, 0.5947(2)	1	0.98(2)

*U*_*ISO*_ displacement parameter, *SOF* site occupancy factor

DTA measurements did not show any detectable signal between 475 and 500 °C; thus, it is currently not possible to give a more exact value of the temperature of the peritectoid reaction (*β*-Bi_2_Rh + BiRh $\rightleftharpoons$ BiRh_0.81_) which is shown tentatively at 480 °C (Fig. 1). The reason for this might be a very small enthalpy effect due to the similar crystal structures of BiRh_0.81_ and BiRh in combination with a very slow kinetics (see below). A consequence of the presence of the BiRh_0.81_ phase is that the *α* $\rightleftharpoons$ *β* transition temperature shifts on the Rh-rich side from 448 ± 2 °C to a somewhat lower temperature. Annealing experiments with samples containing 45 at.% Rh (see Table 3) show that it must be around 440 ± 5 °C. Unfortunately, it is not possible to provide a more precise value of the transition temperature, as no DTA signal could be found in this temperature range.

The detection of BiRh, *β*-Bi_2_Rh and of traces of *α*-Bi_2_Rh in powder XRD and EDX measurements (Fig. 4d and Table 3) showed, that the annealing conditions (440 °C, 27 days) were not sufficient to produce BiRh_0.81_ as a single phase. Together with the fact that more than 2 weeks were necessary to form this compound, it indicates a very slow diffusion and/or formation kinetics. As noted above, the MnP-type (BiRh_0.81_) and NiAs-type (BiRh) structures are closely related and a transformation between them could even be of second order (see, e.g. Kjekshus and Pearson[27] or Franzen et al.[36]). In the current case, however, the orthorhombic lattice parameters deviate considerably from those derived from the hexagonal parameters by cell transformation, pointing rather to a first-order transformation. This considerable distortion is probably due to the reduced occupation at the Rh site in BiRh_0.81_.

### Lattice Parameters of the Phases BiRh and BiRh_0.81_

The unit cell parameters *a*, *c*, the ratio *c/a* and the cell volume *V* of the BiRh phase, measured on powders quenched from 750 °C, are shown in Fig. 7. The lattice parameter *a* increases whereas the lattice parameter *c* decreases with increasing Rh content between 46.9 and 53.0 at.% Rh. The *c/a* ratio decreases in the same direction and lies between 1.391(5) and 1.383(3) which is in the typical range of NiAs phases (but far away from the ratio *c/a* = 1.633, typical for hexagonally close packing). From 42.5 to 46.9 at.% Rh and from 53.5 to 60 at.% Rh the unit cell parameters are constant within the error margins. From the lattice parameter *a* of a sample with 46.5 at.% Rh, annealed at 565 and 900 °C, phase boundary values of 50.5 and 48.6 at.% Rh were derived for BiRh at these temperatures, as indicated by dotted lines in Fig. 7. The corresponding data points are marked by diamonds in Fig. 1.

High temperature powder XRD measurements of a sample with 50 at.% Rh, quenched from 438 °C (Table 5 and Fig. 8), yield slightly different lattice parameter values compared to data by Glagoleva and Zhdanov[17] who analyzed a sample quenched from 780 °C. As shown in Fig. 8, the BiRh phase starts to decompose above 475 °C and disappears completely above 650 °C. This is caused by evaporation of Bi from the BiRh phase into the dynamic vacuum of the high-temperature XRD chamber and leads to the appearance of Rh reflexes only. Rh reflexes in the pattern at ambient conditions are due to small amounts of non-reacted Rh. As a consequence of the loss of the BiRh phase it was not possible to reproduce the significant increase of the lattice parameter c at 800 °C described by Ross and Hume-Rothery.[9]

**Fig. 10:**
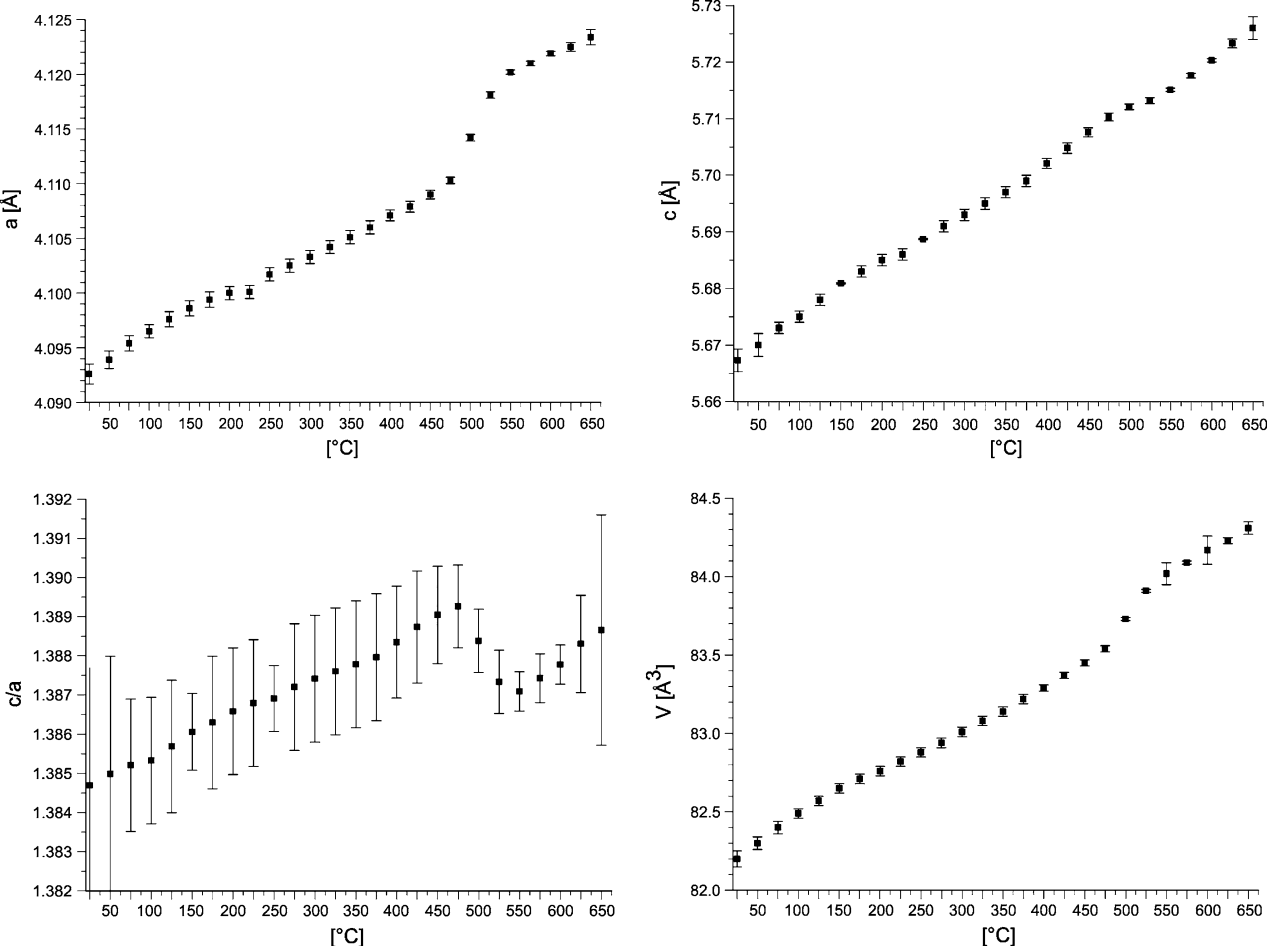
BiRh lattice parameters *a*, *c*, *c/a*, *V* of a sample with 50 at.% Rh, originally quenched from 438 °C, as a function of temperature

With increasing temperature, the lattice parameters *a* and *c* and the volume of the unit cell *V* increase (Table 5 and Fig. 10) for the BiRh phase. In contrast to lattice parameter *c*, which increases in the error margin linearly from 5.667(1) Å at room temperature to 5.726(2) Å at 650 °C, lattice parameter *a* shows a steep increase between 475 and 550 °C [4.110(3)-4.120(2) Å]. This step is probably related to the continuing decomposition of BiRh in the dynamic vacuum which becomes significant at this temperature. The loss of Bi shifts the composition of BiRh to the Rh rich side connected with an increase of lattice parameter *a*.

**Fig. 11:**
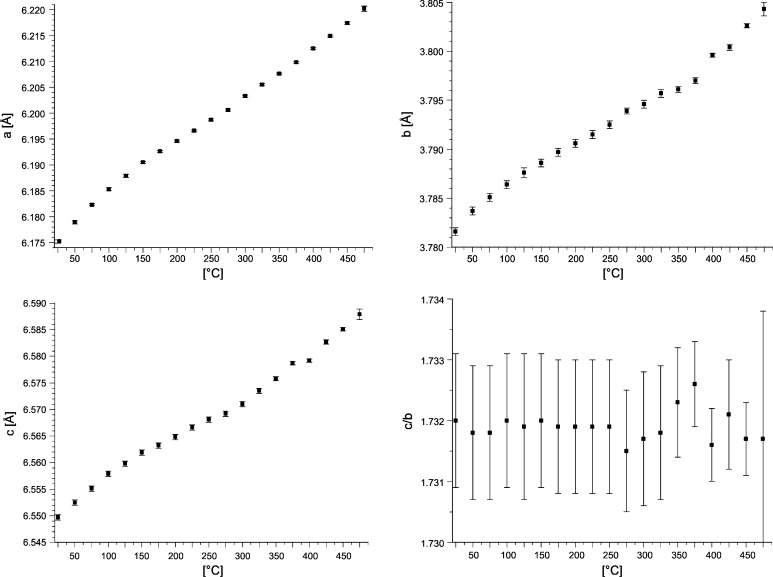
BiRh_0.81_ lattice parameters *a*, *b*, *c*, *c/b*, of a sample with 50 at.% Rh, originally quenched from 438 °C, as a function of temperature

Similar to BiRh, the lattice parameters *a*, *b*, and *c* of the BiRh_0.81_ phase (Table 5, Fig. 11) increase linearly with increasing temperature, too. Surprisingly, the *c/b* ratio does not change and remains more or less constant within the error margin at 1.732 Å, which is not the case in most other MnP-type structure compounds (see, e.g., Selte and Kjekshus[37]).

## Summary

The Bi-Rh phase diagram in the range from 20 to 60 at.% Rh was reinvestigated by standard experimental methods, and a revised version is presented in Fig. 1. Most important result is the discovery of a new phase BiRh_0.81_ that is formed by a peritectoid reaction (*β*-Bi_2_Rh + BiRh $\rightleftharpoons$ BiRh_0.81_) at about 480 °C. Apparently, its existence had been missed up to now due to its very sluggish formation.

The existence of the three phases Bi_4_Rh, Bi_2_Rh (in two modifications depending on temperature), and BiRh could be confirmed. On the other hand, the phase Bi_3_Rh, which had been included in the stable phase diagram by Okamoto,[26] is thought to be actually metastable, as it could never be obtained in samples annealed in the corresponding temperature range. Based on the compositional variation of the lattice parameters, the homogeneity range of BiRh was established in the high-temperature range from about 600 °C up to its peritectic decomposition at 979 °C.

Since the composition range below 20 at.% Rh was not investigated in the present study, nothing can be said about the reportedly metastable compound Bi_14_Rh_3_.

The temperatures of many of the invariant reactions depend on the presence or absence of the two phases Bi_3_Rh and BiRh_0.81_. For example, the temperature of the peritectoid decomposition of *α*-Bi_2_Rh differs by more than 10 °C, depending on the presence or absence of the new phase BiRh_0.81_. Nevertheless, it is thought that the equilibrium diagram is correctly reflected by Fig. 1.

## Footnotes

1 FIZ Karlsruhe, 76344 Eggenstein-Leopoldshafen, Germany, (fax: (49) 7247-808-666; e-mail: crysdata@fiz.karlsruhe.de).
